# Gene Expression Profiling of Lymph Node Sub-Capsular Sinus Macrophages in Cancer

**DOI:** 10.3389/fimmu.2021.672123

**Published:** 2021-06-08

**Authors:** Danilo Pellin, Natalie Claudio, Zihan Guo, Tahereh Ziglari, Ferdinando Pucci

**Affiliations:** ^1^ Gene Therapy Program, Dana-Farber/Boston Children’s Cancer and Blood Disorders Center, Harvard Medical School, Boston, MA, United States; ^2^ Department of Otolaryngology – Head and Neck Surgery, Oregon Health and Science University, Portland, OR, United States; ^3^ Department of Cell, Developmental & Cancer Biology, Oregon Health and Science University, Portland, OR, United States; ^4^ Program in Cancer Biology, Oregon Health and Science University, Portland, OR, United States

**Keywords:** cancer, lymph nodes, macrophages, gene expression, immune system, Fc receptors

## Abstract

Lymph nodes are key lymphoid organs collecting lymph fluid and migratory cells from the tissue area they survey. When cancerous cells arise within a tissue, the sentinel lymph node is the first immunological organ to mount an immune response. Sub-capsular sinus macrophages (SSMs) are specialized macrophages residing in the lymph nodes that play important roles as gatekeepers against particulate antigenic material. In the context of cancer, SSMs capture tumor-derived extracellular vesicles (tEVs), a form of particulate antigen released in high amounts by tumor cells. We and others have recently demonstrated that SSMs possess anti-tumor activity because in their absence tumors progress faster. A comprehensive profiling of SSMs represents an important first step to identify the cellular and molecular mechanisms responsible for SSM anti-tumor activity. Unfortunately, the isolation of SSMs for molecular analyses is very challenging. Here, we combined an optimized dissociation protocol, careful marker selection and stringent gating strategies to highly purify SSMs. We provide evidence of decreased T and B cell contamination, which allowed us to reveal the gene expression profile of this elusive macrophage subset. Squamous cell carcinomas induced an increase in the expression of Fc receptors, lysosomal and proteasomal enzymes in SSMs. Imaging of mouse and patient lymph nodes confirmed the presence of the top differentially expressed genes. These results suggest that SSMs respond to tumor formation by upregulating the machinery necessary for presentation of tumor particulate antigens to B cells.

## Introduction

Lymph nodes are secondary lymphoid organs collecting lymph fluid and migratory cells from the tissue area they survey. When carcinogenic insults hit cells within a tissue, its sentinel lymph node is arguably the first immune organ to detect the accident and to elaborate an immune response. Within lymph nodes, immune cells are highly organized into different anatomical compartments, and such architecture underlies its function (1). Afferent lymphatic vessels deliver lymph-bound antigens and migratory cells into the lymph node sub-capsular sinus. Here, a mesh of cells, mainly macrophages, dendritic cells and lymphatic endothelial cells, filter lymph-bound antigens according to their size. Small, soluble antigens seep through lymphatic conduits and are channeled toward lymph node resident dendritic cells in the paracortical region. On the other hand, large and particulate antigens (>5nm in hydrodynamic radius) are retained by sub-capsular sinus macrophages (SSMs) (2, 3). These specialized macrophages play important roles as gatekeepers against invading pathogens and in relaying immune complexed antigens to B cells for deposition on follicular dendritic cells (4–7). In the context of cancer, SSMs capture tumor-derived extracellular vesicles (tEVs), a form of particulate antigen released in high amounts by tumor cells and overflowing into sentinel lymph nodes very early during disease progression (8).

We have recently demonstrated that SSMs possess anti-tumor activity because in their absence tumors grow faster (8). These observations have been confirmed in additional tumor types (9, 10). However, the cellular and molecular mechanisms responsible for SSM anti-tumor activity are still unknown. In our studies, we unveiled a link between tEVs, SSMs and immunoglobulins. By capturing tEVs, SSMs prevented extensive tEV-B cell interactions, which were required for tumor promotion. Accordingly, increases in lymph node plasma cells and immunoglobulin binding to tEVs was observed. Still, many details about SSMs’ contribution to the generation of humoral immune responses against tumor antigens are missing. How do SSMs capture tEVs? Which signals SSMs release after capturing tEVs? How do they process tEV-bound antigens? A comprehensive profiling of SSMs represents an important first step to answer the above questions and to start elucidating their anti-tumor activity and their contributions to humoral immune responses in cancer.

The isolation of SSMs for molecular analyses is very challenging. Likely due to their role as lymph node gatekeepers, SSMs are highly dendritic in shape and constantly extend and retract pseudopods, likely to survey the sub-capsular space (unpublished live imaging observations). These sub-cellular structures can break apart during conventional enzymatic and/or mechanical dissociation of lymph node tissues, a required step to prepare the samples for flow cytometric analyses and isolation. Consequently, gating strategies that identify SSMs based on surface markers alone can lead to a group of flow cytometric events with odd scatter characteristics. In addition, these SSM-derived blebs can end up sticking to other cells, mainly lymphocytes (11). Indeed, initial attempts to profile SSMs (4) showed a relatively high T and B cell contamination. Improved protocols for minimizing SSMs break down and decrease lymphocyte contamination are thus urgently needed to elucidate the biology of these anti-tumor immune cells.

Here, we combined an optimized dissociation protocol, careful marker selection and stringent gating strategies to highly purify bona-fide intact SSMs. We provide evidence of decreased T and B cell contamination, which allowed us to reveal the gene expression profile of lymph node sub-capsular sinus macrophages in cancer.

## Methods

### Tumor Models

The chemically-induced squamous cell carcinoma model MOC2 was purchased from Kerafast. For tumor formation, 5•10^5^ cells in 50ul of PBS were implanted in the flank of C57B/6 male mice (Charles River Labs) intradermally, near the inguinal lymph node, as previously described (8). After 2 weeks, we selected mice with similar tumor size for tdLN and ndLN collection. All procedures were in accordance with OHSU IACUC.

### Enzymatic Dissociation of Lymph Nodes

LNs from 2 different mice were pooled: 2 tdLN and 6 ndLN (contralateral inguinal, axillary, brachial). Dissociation buffer was prepared by dissolving 3mg/ml of Collagenase IV (Worthington), 0.1 U/ml of DNase I (Roche), 2% FBS, pen/strep and L-glutamine in IMDM. Whole LNs were incubated in 5ml of dissociation buffer at 37°C and 225rpm agitation. After 15 minutes, LNs were mechanically dissociated by passing them through an 18G needle syringe at 2ml/second (LNs burst immediately) and put back at 37°C in same dissociation buffer, at 225rpm agitation. After 15 more minutes, the LN cell suspensions were mechanically dissociated by passing them through an 27G needle syringe at 0.5ml/second for 10 times. The LN cell suspension was then filtered on a 100um cell strainer and washed in 50ml of MACS buffer (2mM EDTA, 2.5% BSA in PBS). All centrifugation steps were performed at 500g for 10 minutes and supernatants were checked at the microscope for absence of cells. If significant cell numbers were observed (>0.1•10^6^/ml), supernatants were diluted 1:1 in MACS buffer and centrifuged again. LN cells were resuspended at ~10^8^/ml for staining (~1.5ml) in MACS buffer.

### Flow Sorting and RNA Extraction

All antibodies (and Fc Block) were from Biolegend, and were used at 2ul per 100ul of cell suspension, with the exception of CD45-PC7 and Ly6G-PE, which were used at 1ul per 100ul of cell suspension. Single stain controls were made by pooling a small amount from all samples. Flow sorting was done on a BD Aria and recovered ~10,000 LN macrophages from each group. LN macrophages were then pelleted and lysed in RLT with B-mercapto-ethanol for RNA extraction (RNeasy micro kit), with on column DNAse treatment.

### RNA Sequencing

RNA integrity was determined using the 2100 Bioanalyzer (Agilent). RNA was converted to cDNA with the SMART-Seq v4 Ultra Low Input RNA kit (Takara) and converted into sequencing libraries using the DNA Nano Kit (Illumina). Libraries were profiled on the 4200 TapeStation (Agilent) and quantified using the Kapa Library Quantification Kit (Roche) on a StepOnePlus Real Time PCR Workstation (Thermo). Sequencing was done on a HiSeq 2500 (Illumina). Fastq files were assembled using Bcl2Fastq2 (Illumina) and aligned using STAR aligner (12).

### Gene Expression Analysis

Gene expression analysis was performed on 16 samples. The sample matrix is reported in **Supplementary Table 1**. We analyzed 4 pooled samples (biological replicates), each of them obtained from collecting cells from 2 mice. Every pool has generated 4 samples: CD11c+ (Phenotype=+) and CD11c- (Phenotype=-) cells sorted from Tumor Draining (TD=1) and Non-Draining (TD=0) lymph nodes. Raw sequencing files have been QC and aligned to the reference mouse genome mm10 using STAR workflow (12), obtaining, as a result, the gene-based count matrices. We removed from the data set those genes with a cumulative read count smaller than 10 and those that have been detected (reads count>0) in less than 2 samples, reducing the gene list from the initial 55423 to 20351. Statistical analysis has been performed in R (13), using DEseq2 package (14). Gene counts have been modeled using a negative binomial model, hypotheses tested by likelihood ratio test (LRT) and all p-value have been adjusted for multiplicity using Holm method (15). A description of the model comparisons that have been performed and their motivation follows.

$$C1.\;M1:GeneExpr_{i}=b_{0}+b_{1}Pool+b_{2}TD+b_{3}Phenotype$$

$$M0:GeneExpr_{i}=b_{0}+b_{1}Pool+b_{2}TD$$

This comparison is focused on the identification of genes with a consistent expression modification between phenotypes, taking into account the possible confounding of TD and Pool factors. The complete list of genes is available in **Supplementary Table 2**.

$$C2.\;M1:GeneExpr_{i}=b_{0}+b_{1}Pool+b_{2}Phenotype+b_{3}TD$$

$$M0:GeneExpr_{i}=b_{0}+b_{1}Pool+b_{2}Phenotype$$

With this analysis, we investigated which are the genes with a significant association with TD variable, adjusting for Pool and Phenotype effects. The complete list of genes is available in **Supplementary Table 3**.

### Quantification of B- and T-Cells Contamination

**Figure 1:**
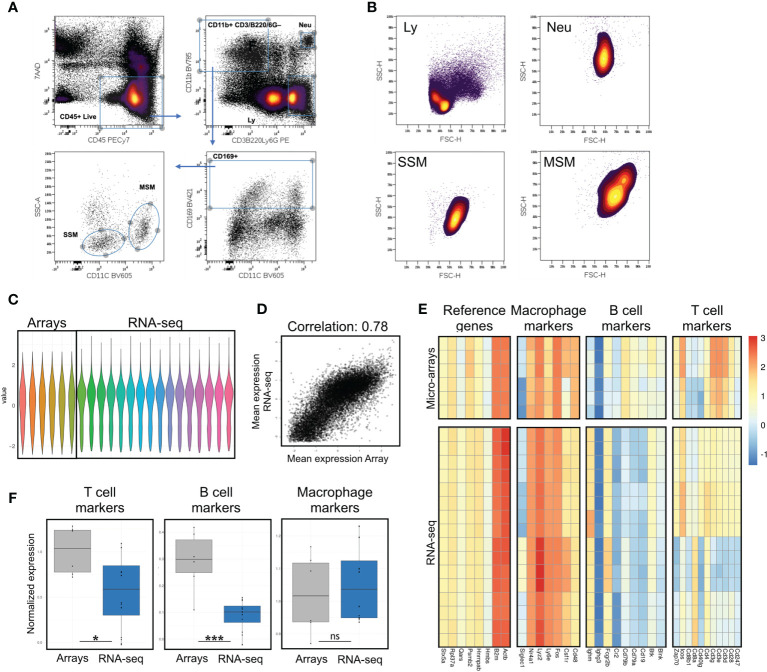
Assessment of potential B and T cell contamination in preparations of lymph node macrophages by comparison with previously published data. **(A)** Sorting strategy; after excluding cell debris in a FSC/SSC plot, live immune cells are gated as 7AAD-CD45+; a stringent gate for the myeloid marker CD11b excludes potential contaminating events with mixed macrophagic/lymphocytic markers, possibly representing SSM-blebs on B/T cells; myeloid cells are then gated for the lymph node macrophage marker CD169; SSMs and MSMs are best separated on a SSC/CD11c plot. **(B)** Forward and side scatter parameters of SSM, MSM, lymphocytes (Ly, gated as in **A**) and neutrophils (Neu, gated as in **A**) are consistent with a macrophagic origin (i.e. bigger and more granular than lymphocytes). **(C)** Normalized values from gene expression arrays and sequencing data. **(D)** Normalization of gene expression arrays and sequencing data preserves high correlation between datasets (Pearson correlation 0.78). **(E, F)** Evaluation of T, B cell and macrophage markers, along with reference genes, shows significant reduction **(F)** of both T and B cell contamination in our sequencing data, as compared to previously published array data. Heatmaps show raw data normalized as in **(C)** Each row is a sample, each column is a gene within the respective panel. Statistical test: Wilcoxon rank sum exact test (*p < 0.05; ***p < 0.001; ns, non-significant).

We quantitatively assessed the B/T-cell fractions in our samples and compared them to previously published data (4) accessible through GEO code GSE15767. RNA-seq and microarray are platforms aimed at measuring gene activity level by mRNA quantification, but they rely on profoundly different techniques and protocols. In order to make the cross-platforms comparison as robust, meaningful, and accurate as possible, we implemented the following analysis workflow. Microarray technology uses several probes distributed along each gene’s mRNA sequence to have a reliable estimate of its expression; hence, we grouped and averaged the expression values based on gene identifier. We filtered out all genes detected only in one platform, reducing the gene list from 55423 to 14003. We normalized the log2 expression values in each sample in the 2 datasets by standardization (subtract mean and divide by standard deviation) and calculate platform-wise gene-specific expression levels by averaging all RNA-seq and microarrays data points. Based on the visual inspection of the scatter-plot in Fig. 1C and on Pearson correlation in Fig. 1D (r=0.78), we concluded that the two datasets are comparable. We defined shortlists of lineage-specific marker genes based on ImmPort gene lists (16):

- B-cell markers: Blnk, Btk, Cd19, Cd79a, Cd79b, Cr2, Fcgr2b, Ighg3, Ighm.
- T-cell markers: Cd247, Cd28, Cd3d, Cd3e, Cd3g, Cd4, Cd40lg, Cd8a, Cd8b1, Icos, Zap70.
- House Keeping: Rpl37a, B2m, Hmbs, Stx5a, Psmb2, Hnrnpab, Actb, Qars.
- Macrophage markers: Csf1r, Cd45, Siglec1, Cd48, Lyz2, Fos, Nr4a1, H2-k1, Ly6e.

For each sample, we calculated the lineage signature values by summing the expression levels of marker genes in the corresponding list (**Figure 1E**). Results are reported in **Supplementary Table 4**. Housekeeping signature has low variability and is used as a reference to determine other lineages contribution. Boxplots of ratios are visible in **Figure 1F** for B/T-cell and Macrophages. Differences in ratio distributions tested using the non-parametric Mann-Whitney test.

### Imaging of Mouse Lymph Nodes

Lymph nodes were fixed overnight at 4°C, rocking, with 2 mL of 4% Paraformaldehyde (Alfa Aesar, 433689M) in PBS. After fixation, any surrounding adipose tissue was carefully dissected under a stereotactic microscope, taking extra care to keep the subscapular sinus intact. Lymph nodes were then stained as previously described (8). Briefly, staining was performed for 24 hours at 4°C, rocking, with the appropriately labeled fluorescent or unconjugated antibodies in 500 µl of Permeabilization/Staining Buffer (0.5% Triton X, 10% Goat Serum, 0.02% Sodium Azide in PBS). Two washes were performed at 4°C, rocking, within a 24 hour period (over day and overnight) in 2 mL of Permeabilization/Staining buffer. The staining and washing procedure was then repeated to stain with fluorescently labeled secondary detection reagents. The antibodies used for staining were: CD169 (3D6.112), FCϵR1a (MAR-1), CD16.2/FCλRIV (9E9), CD64/FCλR1 (OTI1A8), CCL4/MIP-1 (EP521Y) and MMP-14/MT1-MMP (3-F7). After the staining procedure, lymph nodes were fixed again for 2 hours (using fixation conditions mentioned above). For clarification of tissue, lymph nodes were transferred to 5 mL of Cubic Reagent 1 buffer [25% urea, 25% N,N,N’,N’-tetrakis-(2-hydroxypropyl)-ethylenediamine, 15% Triton-X in dH_2_O (17)] and incubated for 3 days, rocking, at 37°C. Following tissue clarification, refractive index matching was performed by placing lymph nodes in 1 mL of Easy Index Optical Clearing Solution (Live Canvas Technologies, EI-Z1001) for 12-24 hours, rocking at 37°C. A wash or “buffer exchange” was performed before each incubation step into a different buffer. All buffers were filtered at each step to avoid deposits of lint/debris onto lymph nodes. On the day of imaging, lymph nodes were mounted in Easy Index between two coverslips using silicon spacers and imaged on a Zeiss/Yokogawa CSU-X1 spinning disk confocal. For all lymph nodes, 20x z-stacks of both the subcapsulary sinus and medullary sinus region were obtained using the same laser power and pixel dwell time. The use of two coverslips allowed to flip the samples under the microscope for imaging both the subcapsulary sinus and medullary sinus. Image stacks were analyzed in Fiji. Pre-processing included denoising (despeckle), stack equalization and background subtraction (roll=30). Ten optical sections for a total depth of 4.4um were then Z-projected (max intensity). For quantification of co-localization signals, Pearson correlation was measured using the Coloc2 plugin.

### Imaging of Patient Lymph Nodes

Formalin-fixed paraffin embedded (FFPE) tissue sections (5µm) of tumor free lymph nodes from anonymized patients (n=2) diagnosed with squamous cell carcinoma of the head and neck were obtained from the Oregon Health and Science University (OHSU) Knight Cancer Institute Biolibrary (IRB number 19903). Sequential immunohistochemical staining (multiplex IHC) was performed on these sections as described previously (18). Briefly, sections were baked, deparaffinized, stained with primary antibody (see **Table 1**, and then with a species specific secondary antibody-labeled polymer conjugated to horseradish peroxidase (Nacalai, USA and R&D Systems). Slides were visualized using an AEC (3-amino-9-ethylcarbazole) HRP substrate (Vector Laboratories, SK-4200) followed by whole slide digital scanning. Secondary antibodies were either inactivated using a peroxidase and alkaline phosphatase blocking reagent (Agilent Technologies, S200389-2) followed by staining of a primary antibody of a different species as described above, or antibodies were stripped using heated citrate buffer (Abcam, ab93678) for subsequent antibody staining of the same species. Scanned images were analyzed in Fiji by first registering images from the same tissue section with Register Virtual Stack Slices plugin, using rigid registration and a minimum and maximum image sizes of 1000 and 4000, respectively. AEC signal was extracted from the yellow channel after processing the images with the RGB to CMYK plugin. Pseudo-color dark field images were generated to show colocalization.

**Table 1:** Antibodies used on patient samples.

Antibody	Manufacturer (Catalog Number)	Dilution	Incubation time (min) Room temp.
Fc gamma RI/CD64	Novus Biologicals (NBP2-45625)	1:500	60
Fc gamma RIIIA/CD16a	Novus Biologicals (NBP2-99278)	1:2000	30
Siglec-1/CD169	R&D Systems (AF5197)	1:200	30
MMP-14/MT1-MMP	Novus Biologicals (NBP2-67415)	1:500	30

## Results

SSMs can be distinguished by other lymph node macrophage populations based on surface markers. In order to select the combination of markers and scatter parameters best suited for the isolation of intact lymph node macrophage subsets, we reasoned that since SSMs may possess low phagocytic activity (4), they may display low side scatter during flow cytometry-based analyses. Our recent work confirmed this hypothesis (8) and in this study we took advantage of this property to purify SSMs from tumor-draining lymph nodes (tdLNs) as 7AAD– CD45+ CD3– B220– Ly6G– CD11b+ CD169+ CD11c– SSC^LO^ (**Figure 1A**). The combination of the above markers provides maximum separation between macrophage populations and excludes unwanted contaminating cells and/or flow cytometric events with mixed markers. As reference macrophage population, we isolated medullary sinus macrophages (MSMs) as 7AAD– CD45+ CD3– B220– Ly6G– CD11b+ CD169+ CD11c+ SSC^HI^. Non-draining lymph nodes (ndLNs) were also collected from the contralateral flank of mice carrying a squamous cell carcinoma model (MOC2), for a total of 4 macrophage subsets. Importantly, SSMs and MSMs gated as above were bigger and had higher side scatter than lymphocytes, which is consistent with a macrophagic origin and suggests that what we are isolating are not small SSM-derived blebs (**Figure 1B**). These results indicate that the combination of markers and scatter parameters we adopted, combined with a modified lymph node dissociation protocol (**Supplementary Figure 1**), can be employed to identify bona-fide lymph node macrophage subsets.

In order to identify the gene signature of SSMs in cancer, we first confirmed our isolation protocol works as expected also on a flow sorter (see *Methods*). After obtaining highly purified lymph node macrophage subsets (~10 thousand cells per sample), we performed deep sequencing on total RNA extracted from sorted cells.

Initial attempts to profile lymph node macrophage subsets (4) showed a relatively high T and B cell contamination. The same authors subsequently identified in IL7ra+Ccr6+ IL17-committed innate-like lymphocytes as a major contaminant in SSM preparations and/or analyses (11). In order to assess potential lymphocyte contamination, we defined 4 gene signatures, one for macrophages, one for B cells, one for T cells and one consisting of reference genes. Before comparing these 4 gene signatures between our gene expression profiling data and previously published microarray data (GSE15767), we normalized the dataset to allow inter-platform comparison (**Figure 1C**). Notwithstanding limitations of such comparisons, the correlation between the normalized values was very good (**Figure 1D**). We observed that the reference genes and the macrophage signatures remained constant between the datasets, whereas the T and B cell signatures were significantly lower (p=0.0266 and p=0.0008, respectively) in our dataset (**Figures 1E, F**). Furthermore, to assess potential contamination of IL7ra+Ccr6+ IL17-committed innate-like lymphocytes, we compared the normalized expression of IL7ra and Ccr6 between published microarray data and our lymph node macrophage preparations. We observed a significant decrease in those two markers in our RNAseq dataset (**Supplementary Figure 2**). These results indicate that the isolation procedure adopted decreased lymphocyte contamination in our lymph node macrophage preparations and that the resulting gene expression data is suitable for investigating SSM biology at the molecular level.

**Figure 2:**
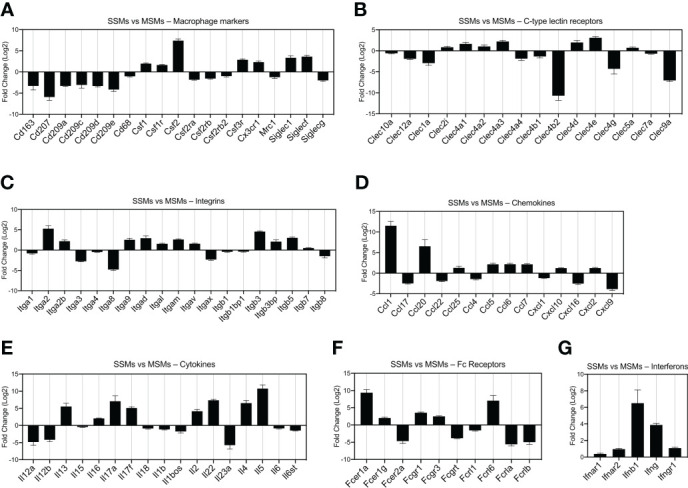
Comparison of gene expression between SSMs and MSMs isolated from non-draining lymph nodes. Gene families of interest are shown. Genes shown have a p-adjusted value < 0.01 and a mean expression level > 100 reads. **(A)** Macrophage markers. **(B)** C-type lectin receptors. **(C)** Integrins. **(D)** Chemokines. **(E)** Cytokines. **(F)** Fc receptors. **(G)** Interferons and receptors. Fold change > 0 indicates genes upregulated in SSMs. Fold change < 0 indicates genes upregulated in MSMs.

To start exploring the gene profile of SSMs, we started from non-tumor draining lymph nodes and compared them to MSMs. We focused on several gene families of interest, including general macrophage markers (**Figure 2A**), C-type lectin receptors (**Figure 2B**), integrins (**Figure 2C**), chemokines and cytokines (**Figures 2D, E**), immunoglobulin Fc receptors (**Figure 2F**) and interferon family members (**Figure 2G**). As expected, SSMs expressed lower levels of the defining marker CD11c (Itgax; **Figure 2C**). To avoid considering genes expressed at very low to no levels, we first filtered for a mean expression (base mean) > 100 reads. Among the genes most highly expressed by SSMs (p adjusted < 0.01), as compared to MSMs, were Csf2, Itga2, Ccl1, Ccl20, Il5, Fcer1a, Fcrl6 and Ifnb1. Among the genes most highly expressed by MSMs (p adjusted < 0.01), as compared to SSMs, were Cd207, Clec4b2, Clec9a, Itga8, Cxcl9, Il12a, Il23a, Fcer2a and Fcrla. These results indicate that SSMs and MSMs possess a unique gene signature that may underlie their specialized functions.

**Figure 3:**
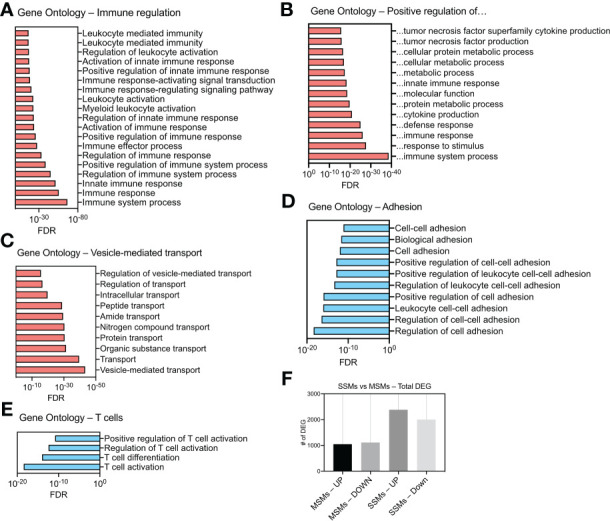
Gene ontology analysis of differentially expressed genes (DEG) in SSMs between tumor-draining and non-draining lymph nodes. **(A)** Gene Ontologies related to immune regulation. **(B)** Gene Ontologies related to positive regulations. **(C)** Gene Ontologies related vesicle-mediated transport. **(D)** Gene Ontologies related to cell adhesion. **(E)** Gene Ontologies related to T cell biology. Gene Ontologies enriched in genes up-regulated between SSMs from tumor-draining and non-draining lymph nodes are shown in red. Gene Ontologies enriched in genes down-regulated between SSMs from tumor-draining and non-draining lymph nodes are shown in blue. Genes with a p-adjusted value < 0.01 and a mean expression level > 100 reads were used. **(F)** Total number of up-regulated and down-regulated DEG in the two macrophage subsets between tumor-draining and non-draining lymph nodes.

We next asked how the presence of a tumor influences SSMs global gene expression. To this end, we compared SSMs profiles between tumor-draining and non-draining lymph nodes and performed gene ontology analysis on up-regulated and down-regulated genes. The results show which biological processes are affected by the presence of squamous cell carcinomas growing in the tissue drained by the local sentinel LN. We observed an increase in expression of genes regulating immune responses and vesicular transport (**Figures 3A–C**). In particular, we found a significant enrichment of genes associated with innate immune responses and immune effector functions (**Figure 3A**). Among gene ontology categories describing positive regulation of cellular processes, we found an enrichment for genes related to cytokine production, protein metabolism and innate immune response (**Figure 3B**). These results suggest that SSMs may have an active role in responding to lymph-borne tumor antigens. Interestingly, we noticed that ontologies related to vesicle-mediated transport were also enriched in SSMs exposed to tumors (**Figure 3C**). On the other hand, we observed a decrease in expression of genes involved in cell adhesion and T cell regulation (**Figures 3D, E**). These additional results suggest that cancers may shape SSM biology by promoting vesicle-mediated transport of lymph-borne particulate antigens, which may be achieved by a decrease in cell adhesion, thereby allowing increased activity of membrane processes. Overall, SSMs displayed twice as many differentially expressed genes as compared to MSMs (**Figure 3F**), further highlighting a preferential influence of tumors on SSMs.

**Figure 4:**
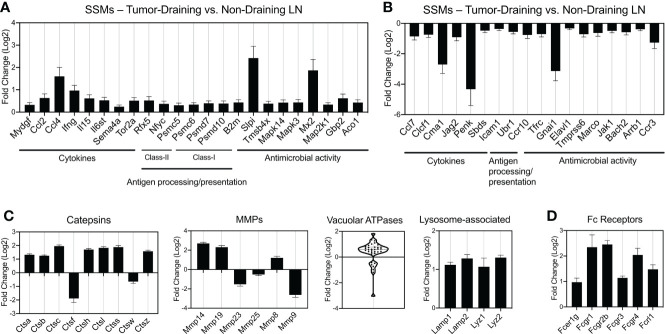
Gene families differentially regulated by tumors in SSMs. **(A, B)** Immune gene lists from the ImmPort project: cytokines (including chemokines, interferons, interleukins, TNF- and TGFb-family members), genes involved in antigen processing and presentation (class-I and class-II restricted) and genes involved in innate immunity, generally annotated as anti-microbial activity genes. Up-regulated **(A)** and down-regulated **(B)** genes are shown. **(C)** Curated gene families for lysosomal genes (Catepsins, vacuolar ATPases and other lysosome-associated genes) and matrix metalloproteases (MMPs). **(D)** Fc receptor genes differentially regulated by tumors in SSMs. All comparisons are between SSMs from tumor-draining lymph nodes versus SSMs from non-draining lymph nodes. Genes with a p-adjusted value < 0.01 and a mean expression > 100 were used.

We demonstrated that SSMs possess anti-tumor activity by showing that in their absence tumors grow faster (8). These observations have been confirmed in additional tumor types (9, 10). The analyses above start to narrow down which potential biological processes may be at the root of SSM biology in cancer. In order to identify the specific molecular alterations associated with SSM anti-tumor activity, we sought to define which specific genes are touched by the presence of tumor cells in the tissue drained by the lymph node where SSMs reside. To this end, we compared SSM gene expression between tumor-draining and non-draining lymph nodes and focused on gene lists of interests (**Figure 4**). We started from gene lists from the ImmPort project (16), which included cytokines (including chemokines, interferons, interleukins, TNF- and TGFb-family members); genes involved in antigen processing and presentation, both class-I and class-II restricted; and genes involved in innate immunity, generally annotated as anti-microbial activity. We observed that among the genes whose expression was most increased by tumors in SSMs were the cytokines Ccl4 and Ifng; the protease inhibitor Slpi and the interferon-induced GTP-binding protein Mx2 (**Figure 4A**). On the other hand, we found that the chymotryptic serine proteinase Cma1, the endogenous opioid polypeptide hormone Penk, Guanine nucleotide-binding protein Gnai1 and the chemokine receptor Ccr3 were among the most down-regulated genes in SSMs after the influence of a local tumor mass (**Figure 4B**). These results suggest that SSMs may respond to lymph-borne tumor material by promoting changes in the local extracellular matrix within the lymph node sub-capsular sinus.

At steady state, SSMs express low levels of genes coding for lysosomal enzymes (4). In order to investigate whether SSM proteolytic activity changes in the context of cancer, we analyzed proteases, vacuolar ATPases and other lysosome-associated genes. Catepsins are proteases that become activated at the low pH found in lysosomes. We found that 7 Catepsin family genes were significantly up-regulated (>2-fold) in SSMs from tumor-draining lymph nodes (**Figure 4C**). When we looked at extracellular proteases, matrix metallo-proteases in particular, we observed that Mmp-14, Mmp-19 and Mmp-8 were also up-regulated in SSMs in cancer (**Figure 4C**). On the other hand, Catepsin-f, Mmp-23 and Mmp-9 were among the highest proteases down-regulated in SSMs from tumor-draining lymph nodes (**Figure 4C**). Vacuolar ATPases acidify a wide array of intracellular organelles, including lysosomes. When we analyzed the family of vacuolar ATPases, we found that the vast majority were up-regulated in SSMs from tumor-draining lymph nodes (**Figure 4C**). In agreement with these findings, we measured an up-regulation of lysosomal markers Lysosomal-associated membrane protein 1 and 2, and Lysozyme 1 and 2 (**Figure 4C**). These results suggest that cancer cells trigger an increase in lysosomal activity in SSMs.

**Figure 5:**
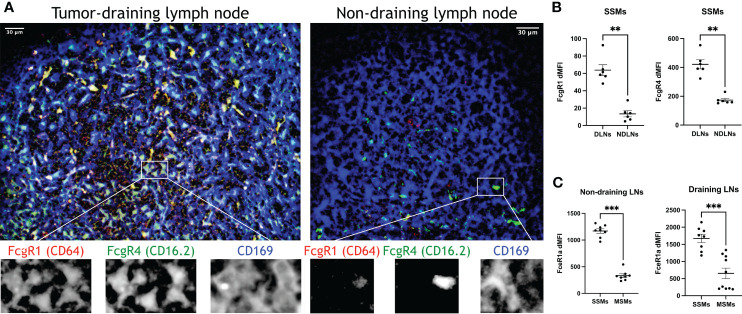
Confirmation of top differentially expressed genes in SSMs. **(A)** Optical section (4.4um) of sub-capsular regions projected from a 3D reconstruction of tumor-draining and non-draining lymph nodes (tdLNs and ndLNs) from mice bearing MOC2 tumors. FcgR1 and FcgR4 were expressed only in SSMs from tdLNs, as compared to SSMs from ndLNs, exceeding the expected upregulation measured by sequencing (**Figure 4D**). Single color details of the highlighted region are shown at the bottom, to confirm co-localization. Images are representative of ~50um-deep 3D volumes (n = 2). Scale bar: 30um. **(B, C)** Flow cytometric analysis of tdLNs and ndLNs from mice bearing MOC2 tumors. As expected (**Figure 4D**), FcgR1 and FcgR4 were upregulated in SSMs from tdLNs, as compared to SSMs from ndLNs **(B)**. As expected (**Figures 2F**, **4D**), FceR1a was upregulated in both ndLNs (left panel) and tdLNs (right panel) SSMs, as compared to MSMs from corresponding lymph nodes **(C)**. Expression intensity (dMFI) was calculated by subtracting intensity of fluorescence minus one controls. SSMs and MSMs were gated as in **Figure 1**. Statistical test by Mann-Whitney test. (**p < 0.01; ***p < 0.001).

The observed increase in proteolytic and lysosomal activity in SSMs from tumor-draining lymph nodes prompted us to interrogate how lymph-borne tumor material may be captured by SSMs. Several studies suggested that tumor cells and tumor antigens form immune complexes with immunoglobulins (19–21). Thus, we asked whether Fc receptors may be involved in capturing tumor material by SSMs. We found that several Fc receptor genes were up-regulated in SSMs from tumor-draining lymph nodes (**Figure 4D**), suggesting that one of the mechanisms by which SSMs capture lymph-borne tumor antigenic material may be *via* Fc receptor binding of immune complexes. In order to further test the tumor-mediated upregulation of Fc receptors in SSMs, we performed imaging and flow cytometric studies of tumor-draining and non-draining lymph nodes. We focused on FcgR1 and FcgR4, because they were both upregulated in SSMs from tumor-draining lymph nodes (**Figure 4D**) and because commercial antibodies were available. We observed that FcgR1 and FcgR4 were almost exclusively found in SSMs from tumor-draining lymph nodes (**Figure 5A**). These findings were confirmed by flow cytometric analysis (**Figure 5B**). In order to test the novel SSM markers identified in our SSM profiling, we performed flow cytometric analysis to compare SSMs and MSMs. We tested FceR1a because it was one of the most highly differentially expressed genes in SSMs (**Figure 2F**). We observed that FceR1a was present at higher levels on SSMs, as compared to MSMs, in both non-draining and tumor-draining lymph nodes (**Figure 5C**). We performed imaging analyses of two additional targets identified as upregulated by tumors in SSMs, MMP14 and CCL4 (**Figure 4**). Although the difference between SSMs from tumor-draining and non-draining lymph nodes was not as stark as in the case of FcgR1 and FcgR4, we measured increased co-localization of MMP14 and CCL4 with CD169+ SSMs in tumor-draining nodes (**Supplementary Figure 3**). Overall, these results indicate that several Fc receptors are upregulated in SSMs by tumors.

**Figure 6:**
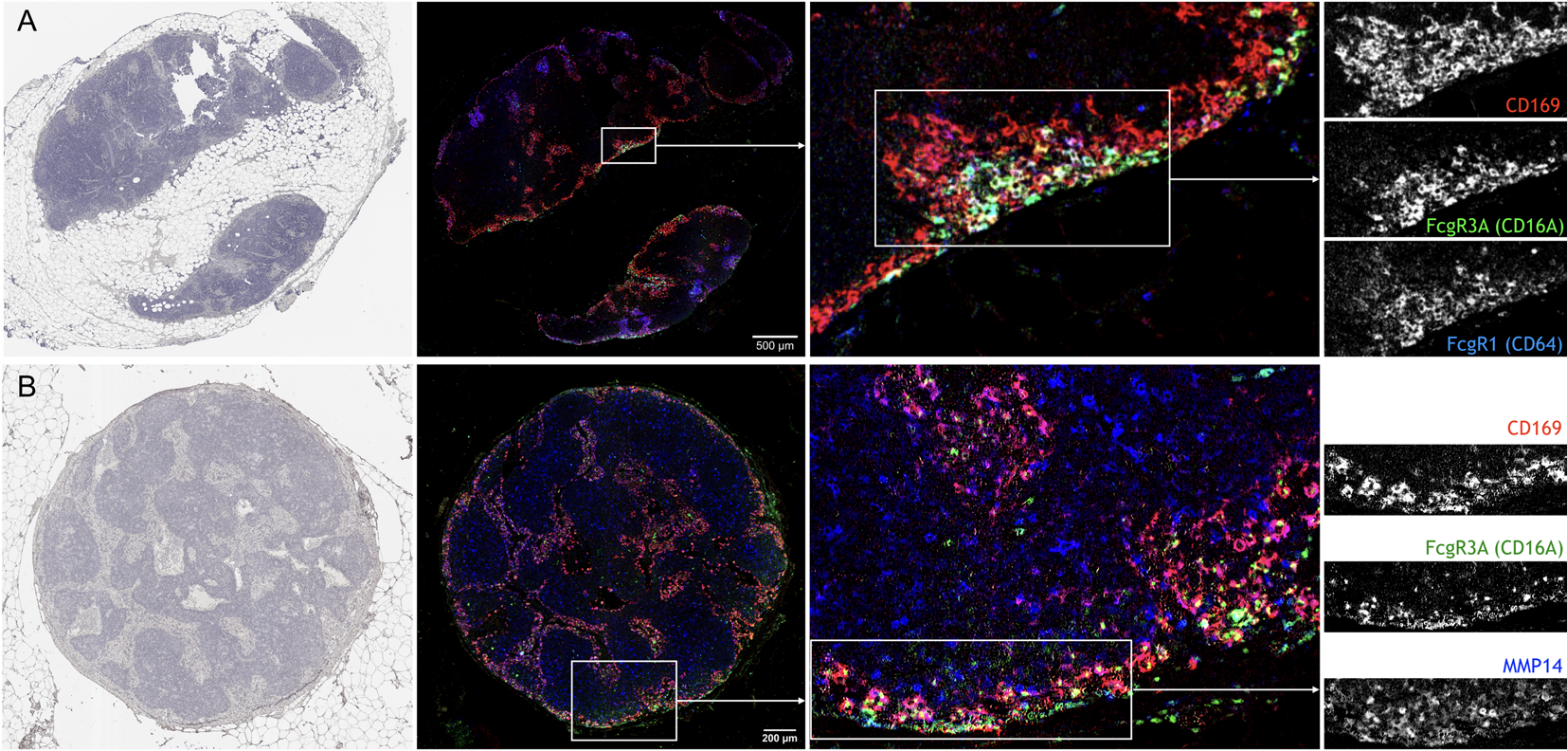
Confirmation of novel SSM markers in regional lymph nodes from head and neck cancer patients with multiplex immunohistochemistry. **(A)** Sequential staining for Hematoxylin (left), CD169, CD16A and CD64 on same lymph node section shows the co-localization of the three markers. Of note, CD169+ macrophages in closer proximity to the sub-capsular sinus were also positive for CD16A and CD64. **(B)** Sequential staining for Hematoxylin (left), CD169, CD16A and MMP14 on same lymph node section shows the co-localization of the three markers. N = 2; Representative pictures are shown.

In cancer patients, the density of SSMs has been associated with increased survival in several different tumor types (22). In order to test whether the markers identified in this study may also apply to SSMs from cancer patients, we performed multiplex immunohistochemistry on regional lymph nodes from head and neck cancer patients. We selected tumor-free, N0 nodes in order to avoid confounding effects of metastatic disease. We observed that FcgR1, FcgR3A (the human equivalent of murine FcgR4) and MMP14 were all present, in different degrees, in human SSMs (**Figure 6**). These observations suggest that the markers we identified in murine models may be applicable to patients as well.

## Discussion

In the past 15 years, lymph node sub-capsular sinus macrophages (SSMs) have been intensively studied in the context of infectious diseases (5–7, 23, 24). However, their roles in different pathological conditions have not been explored as extensively. Our previous work added SSMs as a key cell type in the initiation of immune responses in cancer (8). During those studies, we noticed that flow cytometric analyses were less than ideal for the precise quantification of this specialized macrophage subset. Indeed, SSMs were already known to be sensitive to enzymatic dissociation and tend to tear apart during isolation procedures (11). Lymph node mononuclear phagocytes and reticular cells are best studied by techniques like imaging, that do not compromise cellular integrity (25). This issue makes molecular analyses challenging because SSM blebs (loosely defined as micron-sized membrane-bound cell-derived vesicles carrying parental cell surface markers) stick to other lymphocytes generating flow cytometric events with mixed macrophagic and lymphocytic markers or flow cytometric events smaller than lymphocytes, based on physical scatter. These reasons may explain the fact that, to our knowledge, only one attempt at profiling lymph node macrophage subsets has been done in the past (4). That study employed microarray technology. In this work, we used next generation sequencing to deeply profile highly purified and intact lymph node macrophage subsets in cancer. While this work was under revision, the groups of Drs. Tugues and Detmar published another RNA sequencing based analysis of lymph node macrophage subsets (10). On top of confirming the pro-tumoral activities of SSMs in mammary tumor models, these Authors also suggested that SSMs are profoundly affected by tumor-derived stimuli. Although differences in the isolation procedure may account for the overall limited overlap between our two studies, several of the most highly differentially expressed markers identified in this study were also reported by Drs. Tugues and Detmar, including Fcgr1, Fcer1a, Slpi, Mmp8, Cma1, Ctsc and Lyz2 (**Figure 4**).

By coupling a short and gentle enzymatic dissociation protocol with a stringent gating strategy, we significantly decreased T and B cell contamination in our SSM preparations, as compared to previously reported gene expression studies of lymph node macrophage subsets (4). We employed a different combination of selection markers for flow sorting (i.e. gating both CD169^HI^ and CD169^LO^, and using CD11c/SSC as defining markers instead of F4/80 alone), which provided a better separation between the two CD169+ lymph node macrophage subsets. These advancements allowed us to get a first insight into the biology of SSMs at the molecular level. Considering genes expressed above an arbitrary threshold (mean read count > 100), SSMs expressed higher levels of the following soluble factors, as compared to MSMs (**Figure 2**):

- Csf1 and Csf2, two myelopoietic growth factors
- Ccl1, involved in monocyte recruitment and endothelial cell migration
- Ifng, a key factor in anti-tumor immunity
- Il4, known to signal to B cells and recruited monocytes
- Il5, known to signal to eosinophils
- Il17a, Il17f and Il22, members of the T_H17_ cytokine family

These observations provide a molecular basis for the potential role for SSMs in the above processes. Il4 and Il5, although among the most highly DEG, are expressed at relatively low levels (~200 read counts), and thus it should be determined whether SSMs’ contribution as a source for these cytokines is non-negligible. The T_H17_ cytokines Il17a and Il17f were also expressed at low levels (<1000 read counts), again indicating that our procedure minimized contamination also from Il7ra+ Il17-committed innate-like lymphoid cells [**Supplementary Figure 2** and (11)]. Of interest, SSMs expressed higher levels of Fcgr1 and Fcgr3, two Fc receptors known to preferentially bind activating immunoglobulins (26). This last observation provides a potential molecular basis for the reported anti-tumor activity of these specialized macrophages (8). In this regard, it is worth notice that in cancer, Fc receptor genes were further up-regulated in SSMs from tumor-draining lymph nodes, as compared to SSMs from contra-lateral, non-tumor draining lymph nodes (**Figure 4D** and **Figure 5**). These observations suggest a potential mechanistic basis for SSMs’ ability to capture particulate tumor antigens. Consistent with these observations, specific immunoglobulins from the repertoire of both mice and patients possess high affinity for epitopes on tumor cells (19–21). The functional characterization of immune complexes containing tumor antigens still needs to be defined within the lymph node microenvironment, as does the mechanism by which tumors overhaul these processes to their advantage.

When we compared macrophage subsets between tumor-draining and non-draining lymph nodes, SSM gene profile was affected twice as much as compared to MSM gene profile (**Figure 3F**). This is consistent with the fact that the sub-capsular sinus is the location where tumor-derived particulate material is first encountered (8). The first indication of the effects of squamous cell carcinomas on SSMs was the increase in vesicular transport gene networks (**Figure 3**). In particular, we measured an increase in expression of lysosomal proteases and vacuolar acidifying enzymes (**Figure 4**). Given that lysosomal degradation of extracellular antigens leads to their presentation on major histocompatibility complex (MHC) class-II, these results suggest that SSMs may capture immune complexed tumor antigens *via* Fc receptors and may process them for presentation to B cells.

Pathological conditions such as certain cancers and bacterial invasion of lymph nodes have been demonstrated to decrease SSM density (5, 8). In this work, we found that adhesion processes were down-regulated by tumors (**Figure 3**), along with upregulation of several MMPs (**Figure 4** and **Supplementary Figure 3**), providing a potential mechanistic basis for those observations. Nonetheless, decreased adhesion of SSMs may also suggest the need for increased mobility of those cellular processes tasked with phagocytosing lymph-borne material. The upregulation of CCL4 in SSMs by tumors is of particular interest as the cytokine has been involved in the recruitment of lymph node resident plasmacytoid and type-1 conventional dendritic cells (27). The former may explain how SSMs orchestrate type-I interferon responses (6, 7, 23). Further investigations using *in vivo* imaging approaches able to quantify these activities are needed.

Assessment of regional lymph nodes from head and neck cancer patients confirmed the presence of MMP14 and Fc receptors in SSMs. We noted that MMP14 was also expressed by CD169– cells throughout the paracortical region, and that FcgR1 and FcgR3A were expressed by SSMs only in specific locations and mainly from SSMs with direct access to the subcapsular sinus, which led us to speculate that those SSMs may be closer to tumor-draining afferent lymphatics. More studies are needed to assess the prognostic significance of Fc receptor expression in SSMs.

In conclusion, our work revealed the transcriptome of bona-fide lymph node macrophage subsets in the context of cancer. We identified several biological processes altered in sub-capsular sinus macrophages by squamous cell carcinomas. These data provide the molecular basis for some of the activities previously attributed to sinusoidal macrophages and will further improve our understanding of key pathways at play during sinusoidal immune responses (28). Although these results represent a first step toward a better understanding of the biology of sub-capsular sinus macrophages in cancer, more work is needed to validate additional targets, both as novel biomarkers of lymph node macrophage subsets and from a functional perspective.

## Data Availability Statement

The datasets presented in this study can be found in online repositories. The names of the repository/repositories and accession number(s) can be found below: NCBI [accession: GSE174524].

## Ethics Statement

The animal study was reviewed and approved by OHSU IACUC (2395). Patient sample collection was approved by OHSU IRB (19903).

## Author Contributions

FP conceived the work, FP, NC, TZ and ZG designed the experiments, FP, NC and DP analyzed the data and wrote the manuscript, TZ and ZG reviewed the manuscript. All authors contributed to the article and approved the submitted version.

## Conflict of Interest

The authors declare that the research was conducted in the absence of any commercial or financial relationships that could be construed as a potential conflict of interest.
