# Supplementary figures and images for: Gene Expression Profiling of Lymph Node Sub-Capsular Sinus Macrophages in Cancer
Source: Front Immunol. 2021 Jun 8;12:672123. doi: 10.3389/fimmu.2021.672123 (PMC8218730; doi:10.3389/fimmu.2021.672123)

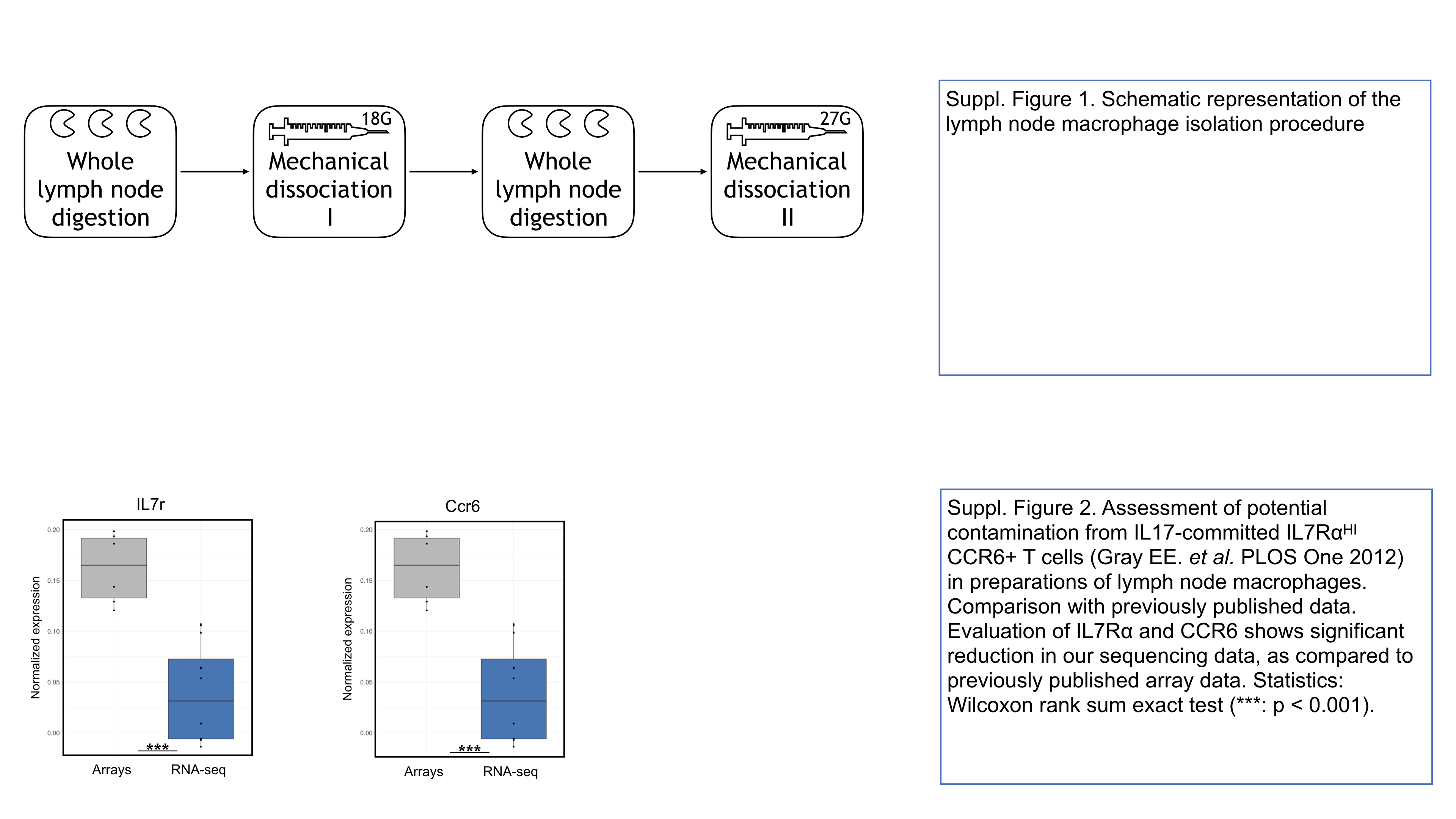

Supplement: Supplementary file 2 [file Image_1.jpeg]

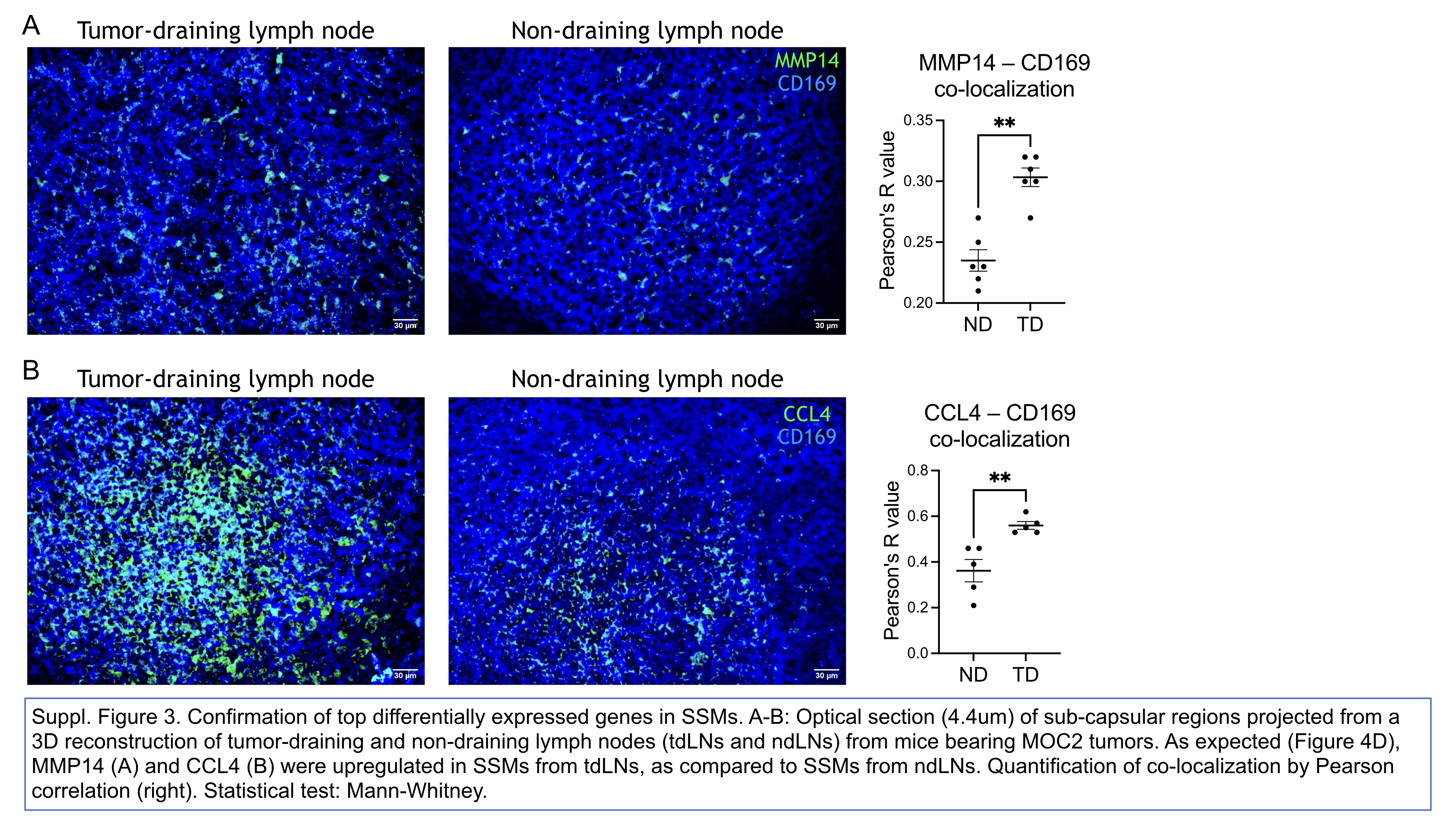

Supplement: Supplementary file 3 [file Image_2.jpeg]
